# Epidemiology of acute gastroenteritis in France from November 2019–August 2021, in light of reported adherence to COVID-19 barrier measures

**DOI:** 10.1038/s41598-022-22317-7

**Published:** 2022-10-19

**Authors:** Athinna Nisavanh, Imene Horrigue, Marion Debin, Clément Turbelin, Charly Kengne-Kuetche, Oriane Nassany, Katia Ambert-Balay, Nathalie Jourdan-Da Silva, Isabelle Pontais, Henriette de Valk, Gabrielle Jones

**Affiliations:** 1 French Public Health Agency, Santé Publique France, Saint-Maurice, France; 2 Sorbonne Université, INSERM, Institut Pierre Louis d’Epidémiologie et de Santé Publique, IPLESP, 75012 Paris, France; 3 National Reference Centre for Gastroenteritis Viruses, University Hospital of Dijon, Dijon, France; 4 ECDC Fellowship Programme, Field Epidemiology Path (EPIET), European Centre for Disease Prevention and Control (ECDC), Solna, Sweden

**Keywords:** Public health, Gastroenteritis

## Abstract

Since the start of the COVID-19 pandemic, French health authorities have encouraged barrier measures and implemented three lockdowns to slow SARS-CoV-2 transmission. We aimed to examine the impact of these measures on the epidemiology of acute gastroenteritis (AGE) in France, from November 2019 to August 2021. We describe trends in AGE indicators from syndromic surveillance and a sentinel surveillance network. Additionally, we describe reported AGE illness data from a community based cohort, and frequencies of adherence to COVID-19 barrier measures from repeated quantitative surveys. From week 7 in 2020, all AGE indicators reached the lowest levels observed since the last decade. During the first lockdown, the median incidence rate reported by the sentinel network was 32 per 100,000 inhabitants, 1.9 times lower than the minimum registered during the 2010–2019 period. Low activity persisted until April 2021. Reported illness from the community cohort mirrored these trends. Adherence to COVID-19 barrier measures was highest during the first lockdown, coinciding with the steep decrease in AGE incidence. Among children under 5 years, AGE incidence increased after the third lockdown in June and July 2021, but remained lower than previous winter-season peaks. Our study indicates that a reduction in adherence to COVID-19 barrier measures, and the end of the lockdowns, coincided with an increase in AGE incidence, particularly among young children. We therefore strongly recommend maintaining adherence to barrier measures in order to in order to limit the transmission of AGE related pathogens.

## Introduction

The COVID-19 pandemic has caused more than 14 million cases and more than 127,000 deaths in France^1^. In March 2020, barrier measures such as mask wearing, handwashing, social distancing were highly encouraged by the national health authorities in order to prevent SARS-CoV-2 infections^2^. A first national lockdown occurred between 17 March to 10 May 2020 [week (W) 12-2020 to W19-2020], and along with barrier measures, schools, bars and restaurants were closed to reduce the burden on the healthcare system. Two additional lockdowns were implemented by the government to reduce the incidence of COVID-19 cases, between 30 October and 15 December 2020 (W43-2020 to W51-2020), and from 3 April to 3 May 2021 (W13-2021 to W18-2021)^2^.

Acute gastroenteritis (AGE) is a common illness, with an estimated 21 million AGE episodes occurring annually in France^3^. A seasonal peak in incidence is usually observed between late December and February, depending on the year^3^. Among children, particularly under 5 years of age, a second seasonal peak usually occurs between February and April^4^. AGE is characterised by diarrhea and/or vomiting, and can be associated with clinical manifestations such as abdominal pain, bloody diarrhea, fever, nausea and headache. The etiology of infectious AGE is most often viral, in particular during the winter season, but AGE can also be caused by bacterial or parasitic infection. The two most frequent viral etiological agents, norovirus and rotavirus, are highly transmissible and drive the AGE winter outbreaks^5^. Gastrointestinal pathogens are transmitted primarily by the fecal–oral route, either directly by contact with an infected person or indirectly, by the ingestion of contaminated food or water, or contact with contaminated objects or surfaces. During the COVID-19 pandemic, barrier measures along with nationwide lockdowns may have had an impact on the transmission of common viruses, other than SARS-CoV-2.

In this study, we aimed to examine the impact of COVID-19 barrier measures and nationwide lockdowns in France on the epidemiology of AGE, from November 2019 until August 2021, in comparison with 2010–2019 historical data.

## Methods

### Data sources and indicators

Data on AGE activity were collected from the routine surveillance databases of the syndromic surveillance system (SurSaUD), and the Sentinel surveillance network of general practitioners (GPs) (Sentinel Network).

Additional data were extracted from the prospective internet based cohort GrippeNet.fr-COVIDnet.fr and the adherence to COVID-19 barrier measures was estimated through data from a repeated quantitative surveys since the start of COVID-19 pandemic (CoviPrev surveys).

SurSaUD, implemented by Santé publique France (SpFrance) since 2004, is based on two morbidity data sources: the emergency department (ED) network, named OSCOUR, and the general practitioners (GPs) emergency house-call associations, named SOS Médecins^6^. The system monitors long-term disease trends and provides early warning of seasonal or unexpected events^7^. It collects daily individual data including administrative and demographic information, and coded medical diagnoses, from more than 700 EDs (92.3% of the national ED attendances), and from 62 of the 63 SOS Médecins associations, covering the whole of France including the overseas regions. OSCOUR defines AGE using one of the 11 codes of the International Classification of Diseases-version 10 (ICD-10), corresponding to AGE of presumed infectious origin (including A090, A099, A09, A084, A083, A080, A085, A081, A091, A082 and A08). For SOS Médecins, AGE is defined by (a) the occurrence of at least three watery stools in less than 24 h, or (b) one to two watery stools with at least one vomiting episode, or (c) vomiting and abdominal pain without other symptoms, excluding chronic gastroenteritis, excessive alcohol ingestion, and symptoms related to pregnancy, menstruation or certain drug treatments. The indicators of AGE activity monitored by these surveillance systems are the estimated weekly proportion of ED attendances for AGE (over the total number of attendances) for OSCOUR, and the weekly proportion of consultations for AGE (over the total number of consultations) for SOS Médecins.

The Sentinel surveillance Network has been monitoring primary care consultations for common illnesses in mainland France for the past 30 years. In 2020, 1275 volunteer GPs were registered in the Sentinel Network, which represent around 2% of all GPs in France^8^.

Each week, the sentinel GPs report the number of patients consulting for AGE (and nine other diseases or medical conditions), based on the following case definition “at least 3 watery or loose stools per day for less than 14 days, motivating consultation”^9^. Data collected on cases are: age, sex, referral for hospitalization after the consultation and, if so, the reason. The data are extrapolated to all GPs in mainland France to estimate the weekly incidence rates (per 100,000 inhabitants) of AGE seen in primary care consultation.

GrippeNet.fr-COVIDnet.fr is an online surveillance system existing since 2012^10^. Every winter, data are collected between November and April–May, except for 2019–2020 when the surveillance was extended until 12 July 2020, because of the COVID-19 pandemic situation. Volunteers living in mainland France can register on the GrippeNet.fr website (www.grippenet.fr) to be included in this prospective community cohort. At registration, they are asked to fill a first intake questionnaire, covering demographic, geographical, socio-economic, and health-related factors. Participants receive a weekly electronic newsletter with a personal link to access the questionnaire, where they report if they have developed symptoms; not only of influenza- or COVID-19-like symptoms, but also of AGE and other health conditions. If they had any symptoms developed since their last connection, they are invited to answer complementary questions, such as the dates of symptom onset and end, care-seeking and self-medication practices. In this surveillance, AGE was defined as having an episode of at least 3 watery or loose stools per day. A symptom-free period of 14 days was used to consider two episodes as distinct. Computation of incidence rates (per 100,000 inhabitants) was adapted from the method previously published for influenza-like illness surveillance^11^. The age structure of the GrippeNet.fr population being different from the general French population, analyses were adjusted by age groups^10–12^. The demographic data of the French population used for adjustment was provided by the French National Institute of Statistics and Economic Studies (INSEE)^12^.

Monitoring of adherence to COVID-19 barrier measures are based on series of repeated quantitative surveys, called CoviPrev, launched by SpFrance since the first national lockdown in March 2020 and carried out at varying frequencies. The surveys are still ongoing at the date of submission of this manuscript. The objectives of CoviPrev are to monitor the adherence to COVID-19 barrier measures, and to assess behavior (alcohol and tobacco consumption, diet and physical activity) and mental health in the general population during the COVID-19 pandemic^13^. The aim of the surveys is to guide and adjust communication on COVID-19 prevention and control measures. Each survey enrolled an independent sample of 2000 people aged above 18 years, living in mainland France, who were asked to fill out an online questionnaire. The sampling was carried out by quotas (sex, age, socio-professional category of the respondent, region, level of urbanisation) adjusted on the 2016 general population census. For our study, we looked at the systematic adherence to four barrier measures that could have an impact on AGE transmission, during the last days preceding the moment of the questionnaire: handwashing (without distinction between using soap and water, or alcohol based hand sanitizer), no handshaking/kissing on the cheek/hugging, avoiding gatherings and remaining at home as much as possible.

All methods were carried out in accordance with relevant guidelines and regulations. Surveillance data collected through SurSaUD and the French Sentinel surveillance Network are regulated by the National Data Protection Agency (CNIL) agreement (respective registration numbers: 1691388 and 471393). SurSaUD and the Sentinel Network provided aggregated anonymous data for which informed consent is not required, therefore, approval from an ethics committee was unnecessary. However, all subjects are informed about the collection of their data for surveillance purposes and they have the possibility to oppose the transmission of their data.

GrippeNet.fr-COVIDnet.fr is conducted in agreement with French regulations on privacy and data collection, and treatment was approved by the Advisory Committee on Information Processing for Research (CCTIRS, authorization 11.565), and also by the CNIL (authorization DR-2012-024). The research protocol of CoviPrev survey was registered by the EHESP School of Public Health Office for Personal Data Protections and approved by the ethical committee of the University Hospital Institute “Mediterranee Infection” (Marseille, France). Participant consent is informed and provided through registration for GrippeNet.fr-Covidnet.fr and CoviPrev survey.

### Analysis

We described the change in the weekly proportions of ED attendances for AGE, the weekly proportion of emergency house-call consultations for AGE, and the weekly incidence rate of GP consultations for AGE, from November 2019 to August 2021, compared to the median, minimum and maximum of the weekly proportion (SurSaUD) and weekly incidence rate (Sentinel Network) on the period from January 2010 to December 2019. Comparisons were carried out for all ages and for children under 5 years of age for OSCOUR and SOS Médecins, and for all ages for Sentinel Network. For GrippeNet.fr/COVIDnet.fr, we compared AGE incidence rates of the 2019–2020 and 2020–2021 winter seasons to those of the 2017–2018 and 2018–2019 winters.

Concerning CoviPrev data, we described the trends in frequencies of systematic adherence to barrier measures against SARS-CoV-2 since the start of the COVID-19 pandemic.

## Results

### Emergency department visits and GP consultations (OSCOUR, SOS Médecins, Sentinel Network)

#### All ages

**Figure 1:**
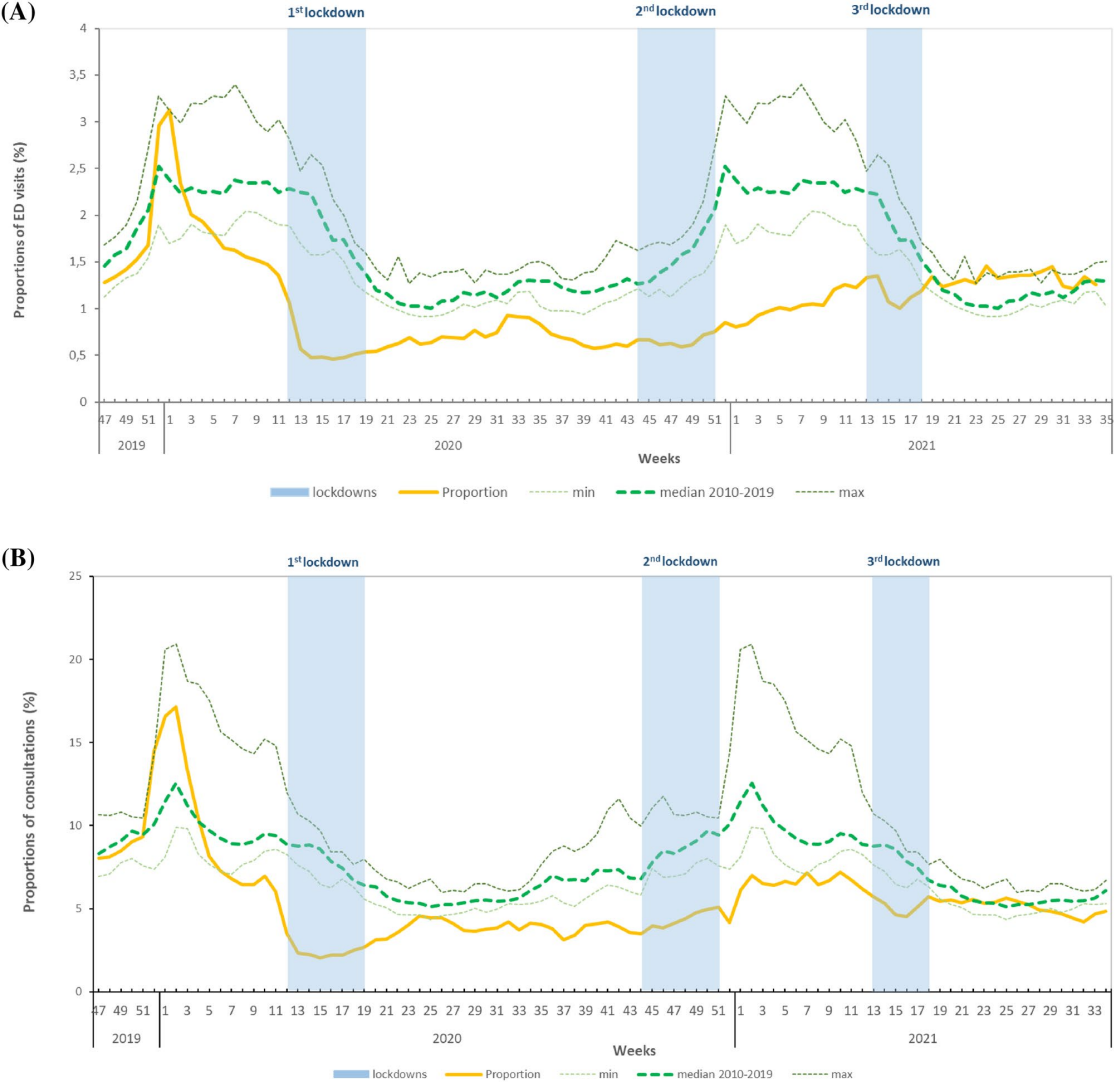

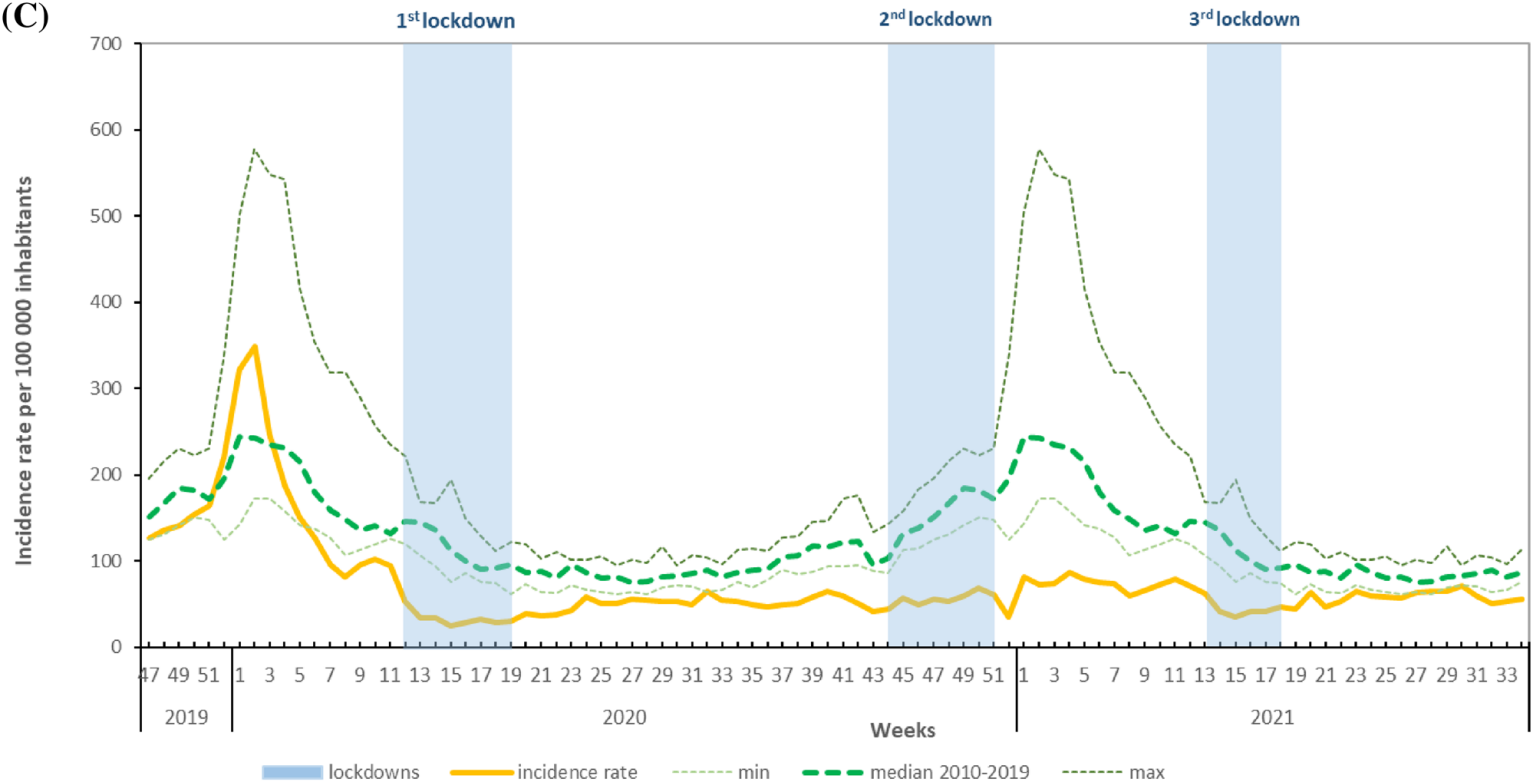
Weekly proportion of consultations for acute gastroenteritis, November 2019–August 2021 (yellow line), in (**A**) emergency departments (OSCOUR), (**B**) GP emergency house-calls (SOS Médecins), and (**C**) weekly incidence rate of GP consultations (Sentinel Network), compared to the minimum, median and maximum in 2010–2019 (dotted lines), all ages, France. Light blue boxes: nationwide lockdowns.

Between W51-2019 and W2-2020, all partners of the AGE Surveillance system registered unusual and very high activities for AGE, reaching a peak in W1-2020 for OSCOUR, and in W2-2020 for SOS Médecins and Sentinel Network (Fig. 1). Such high level of activity had not been observed since the 2010–2011 and 2012–2013 winter seasons. This unusual activity corresponded to a large norovirus outbreak suspected to be linked to the consumption of contaminated raw shellfish^14^. This major peak was followed by a rapid decrease of the activity for all indicators of AGE after W2-2020.

From W7-2020 until W18-2021, and particularly during the first national lockdown (between W12-2020 and W19-2020), the levels registered were lower than the minimum observed during the previous 10 years in all three data sources. During the first lockdown, the median weekly proportion for OSCOUR was 0.50% [range 0.46–1.07%] and for SOS Médecins, 2.30% [range 2.03–3.53%]. These proportions were nearly 2.5 times lower compared to the minimum observed in the 10 previous years at the same period (OSCOUR: median 1.86%, [range 1.18–2.81%], SOS Médecins: median 7.98% [range 5.59–11.99%]). For the Sentinel Network, the median weekly incidence rate was 32 per 100,000 inhabitants [range 25–53], 1.9 times lower than the minimum weekly incidence rate observed during 2010–2019 (median 113 per 100,000 inhabitants [range 61–222]).

By the end of the first lockdown, AGE indicators began to rise slightly and stabilized at levels remaining lower than historic figures: around 0.7% for OSCOUR, 4.0% for SOS Médecins and 53 per 100,000 inhabitants for the Sentinel Network.

No peak in activity was observed during the 2020–2021 winter season in any of the data sources, and the historically lowest levels in AGE activity were registered until April 2021 for OSCOUR and SOS Médecins and until August 2021 for the Sentinel Network (Fig. 1).

### Children under 5 years of age

**Figure 2:**
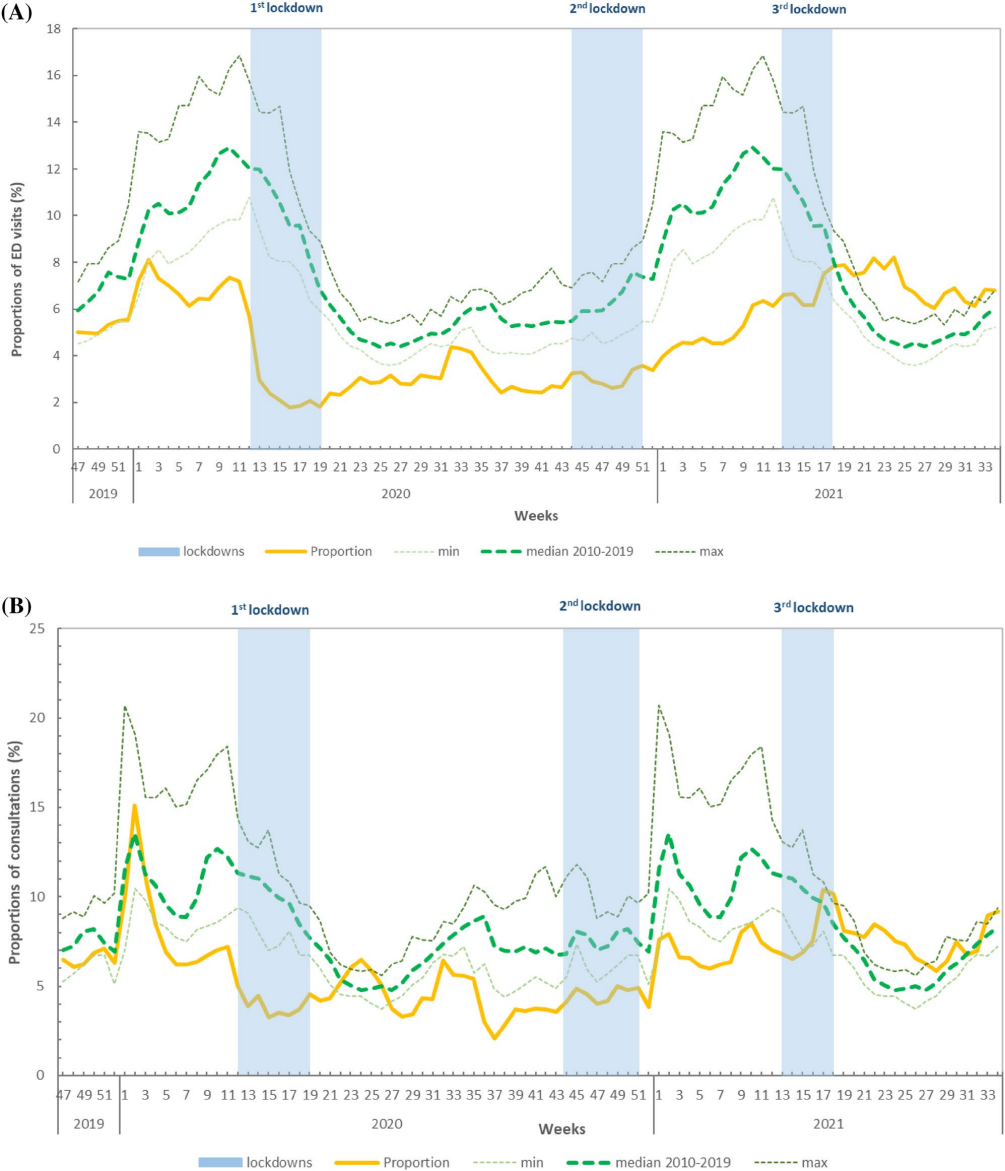
Weekly proportion of consultations for acute gastroenteritis, November 2019–August 2021 (yellow line), in (**A**) emergency departments (OSCOUR), (**B**) GP-emergency house-calls (SOS Médecins), and minimum, median and maximum of these indicators in 2010–2019 (dotted lines), children under 5 years old, France. Light blue boxes: nationwide lockdowns.

For children under 5 years of age, an unusually low level of AGE activity was observed throughout 2020 (Fig. 2A). The median weekly proportion of ED visits was 2.99% [range 1.79–8.12%], lower than the minimum registered in any previous comparable period from 2010 to 2019 (median 6.28% [range 3.60–16.86%]. This low level persisted until mid-March 2021, when a slight increase was observed, surpassing in W12-2021 the levels registered in 2020 at the same period. After W21-2021 the activity for AGE in ED was slightly higher than the maximum registered during 2010–2019 period.

For SOS Médecins, activity levels fluctuated throughout 2020, but remained overall lower than the minimum observed during the 2010–2019 period (Fig. 2B). A peak was reached in W24-2020, small compared to winter-season peaks, but higher than maximum values observed in the summer period over the 10 previous years. The activity then decreased and returned to levels below the minimum observed over the 10 previous years until W16-2021.

From W17-2021, high levels of activity were registered surpassing at times the maximum observed during the 2010–2019 period.

### GrippeNet.fr-COVIDnet.fr cohort

The overall trend of weekly incidence rates for AGE obtained from the cohort was similar to the activity described previously for ED visits, GPs emergency house-calls and GPs consultations.

**Figure 3:**
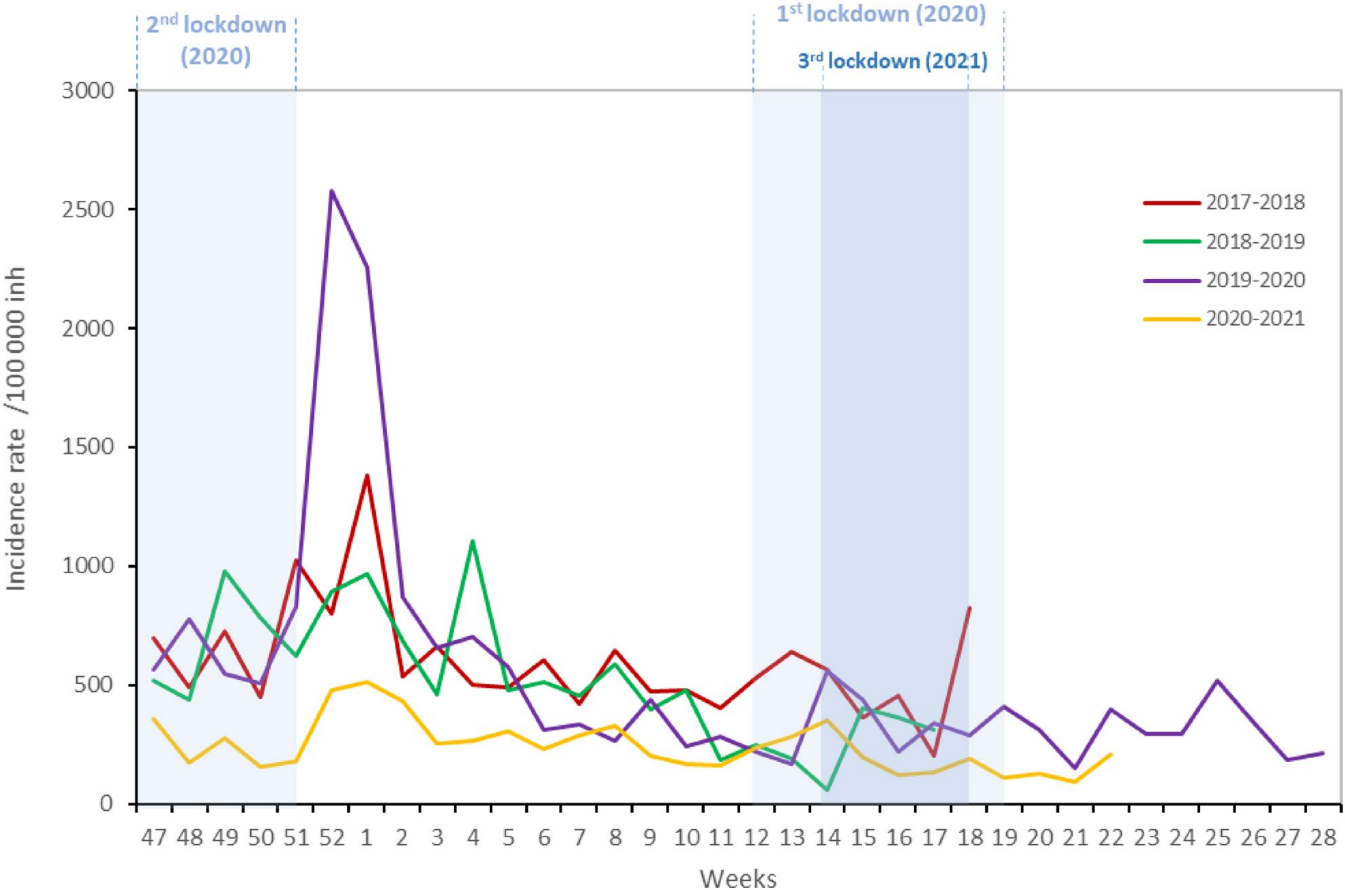
Weekly incidence rates (/100,000 inhabitants) of acute diarrhea from the last 4 winter’ seasons (2017–2018 to 2020–2021). Red line: from W47-2017 to W18-2018, green line: W47-2018 to W17-2019, purple line: W47-2019 to W28-2020, yellow line: W47-2020 to W22-2021, blue boxes: nationwide lockdowns. GrippeNet.fr-COVIDnet, France.

During the 2019–2020 winter season, the unusual peak in activity due to the large norovirus outbreak from the end of December 2019 to early January 2020 was detected in GrippeNet.fr data, with a peak observed in W52-2019 reaching 2579 cases per 100,000 inhabitants [95% CI 1947–3317] (Fig. 3). Following this peak, incidence rates fluctuated from one week to the next and were similar between seasons. When considering the whole winter period from W1 to W17 (period in which data are available for all four seasons), the median weekly incidence rate in 2020–2021 was lower than registered in the three previous winter seasons: 254 per 100,000 inhabitants compared to 535 per 100,000 inhabitants during the 2017–2018 winter season, 479 per 100,000 inhabitants during the 2018–2019 winter season, and 374 per 100,000 inhabitants during the 2019–2020 winter season.

### Adherence to COVID-19 barrier measures: CoviPrev surveys

**Figure 4:**
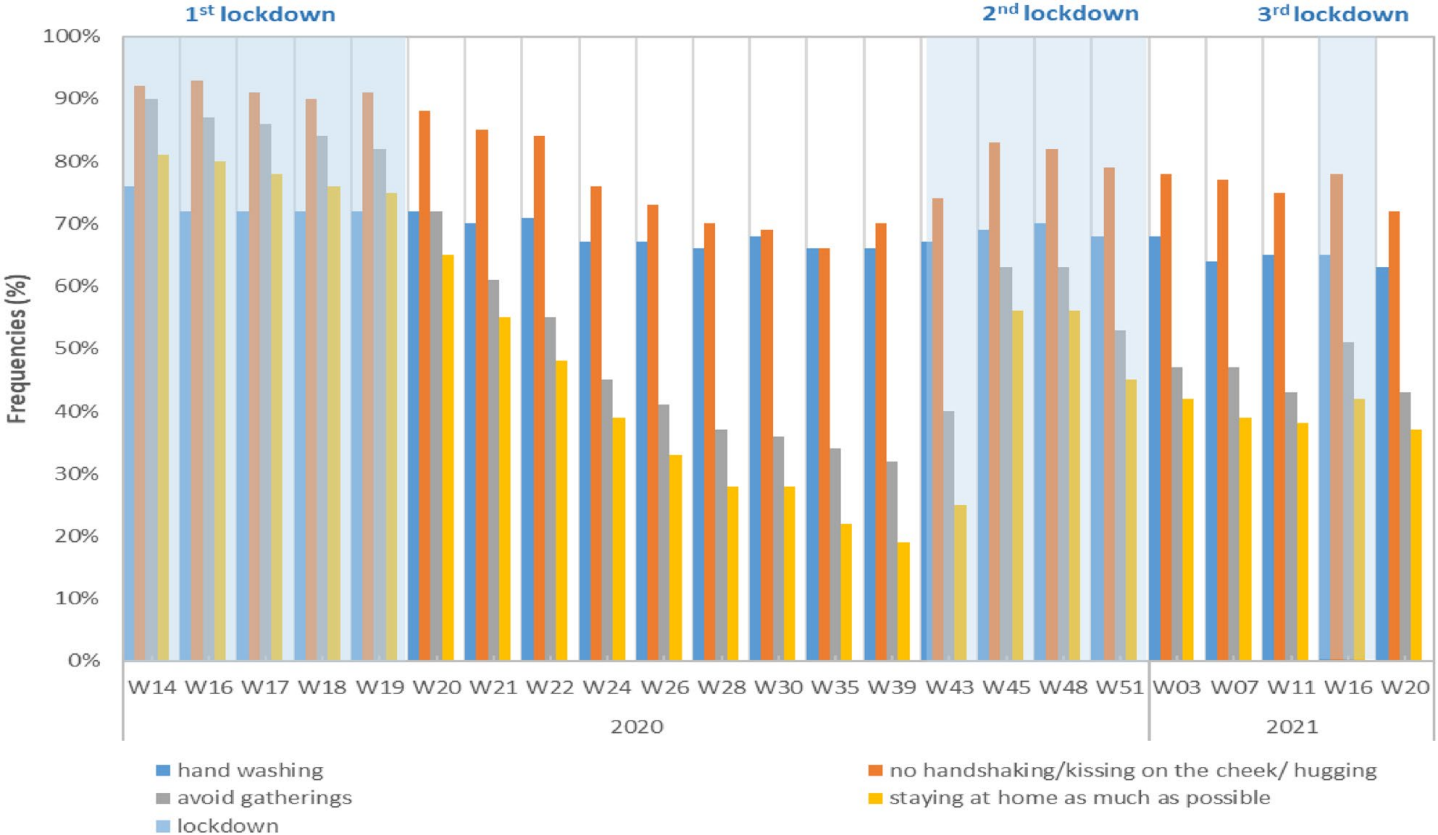
Frequencies of systematic adherence to barrier measures against SARS-CoV-2, CoviPrev survey, metropolitan France, week 14–2020 to week 20–2021 (only weeks with data are displayed). Light blue boxes: nationwide lockdowns.

During the first nationwide lockdown, results of the CoviPrev survey showed a high frequency of adherence to the recommended COVID-19 precautionary measures^15^ (Fig. 4). The median frequency of adherence was 72% for handwashing, 91% for no handshaking/kissing on the cheek/hugging, 86% for avoiding gatherings, 78% for staying at home as much as possible. These frequencies decreased after the first lockdown, reaching between W20-2020 and W43-2020, a median frequency of 67% for handwashing, 74% for no handshaking/kissing on the cheek/hugging, 41% for avoiding gatherings, and 31% for staying at home as much as possible.

During the second lockdown, beginning W43-2020, survey results were rather mixed: the frequency of adherence to systematic handwashing was close to the first lockdown (median 69%). However, the median frequency of adherence for no handshaking/kissing on the cheek/hugging was 82%, 9% lower than during the first lockdown, and for avoiding gatherings and staying at home, the median frequencies decreased by 23% and 22% respectively.

Between the second and third lockdown (from W52-2020 to W12-2021), a slight decrease was again noticed for the adherence to all precautionary measures. During the third lockdown (from W13-2021 to W18-2021), only one survey was carried out, in W16-2021, and the results showed frequencies quite similar to those observed in the pre-lockdown period: 65% for systematic handwashing, 78% no handshaking/kissing on the cheek/hugging, 51% for avoiding gatherings, and 42% for staying at home as much as possible.

These results show an overall decrease over time in the adherence to certain barrier measures, particularly for avoiding gatherings and staying at home. Even if adherence to handwashing and no handshaking/kissing on the cheek/hugging was less frequent after the first lockdown, the overall frequencies for both measures remained relatively high over all the surveys (Fig. 4).

## Discussion

We described the unusual trends in indicators for AGE observed in France during the COVID-19 pandemic, using diagnostic surveillance data from medical consultations (ED visits, GPs emergency house-calls and GPs consultations), and reported illness data from an internet based community cohort (GrippeNet.fr-COVIDnet.fr). Furthermore, we put these trends into perspective and in light of reported adherence to COVID-19 barrier measures from CoviPrev surveys, and nationwide lockdowns. From W7-2020 (February 2020), all indicators for AGE reached the lowest levels observed in 10 years of retrospective data and historically low levels persisted until W17-2021 (April 2021). During the first national lockdown, from 17 March to 10 May 2020, the AGE activity was lower than the minimum observed during the pre-COVID-19 period for ED visits, GPs emergency house-calls and GPs consultations. After W18-2021 (May 2021), all indicators returned to the usual levels observed before the pandemic, and ranged between the minimum and maximum levels registered in historical data. However, among children under 5 years of age, unusually high levels were observed for ED visits and GP emergency house-calls in June and July 2021, compared to previous years at the same period.

Overall decreasing trends in AGE incidence based on French AGE surveillance systems have previously been described in France by Rivière et al. with a decrease in AGE incidence for ED visits since the 2004–2005 season and for the Sentinel Network since the 2008–2009 season. The reasons for this decrease remain unknown^5^. However, it is unlikely that the abrupt and steep decline observed in early 2020, and the persistent low level of activity in 2020 and 2021, could be explained by this long term decreasing trend or by seasonal variations. It could also be argued that the surveillance systems could have been impacted by a massive underreporting of AGE cases. However, this seems unlikely as the surveillance protocols remained the same since the start of the surveillance, and data transmission was automated for ED and GPs emergency house-calls. Another hypothesis to explain the sudden decrease in reported AGE incidence could be possible changes in health care seeking behavior due to the context of the COVID-19 pandemic with a strong reduction of consultations for AGE. However, these surveillance systems include data for other diseases with person to person transmission, including bronchiolitis, seasonal flu and chicken pox and similar trends were observed^16–18^. In contrast, in 2020 and 2021, the reporting rates of Lyme borreliosis, for which there is no person to person transmission, were in the usual range of those observed over the last years^19^ [data from 2021 are not publicly available yet]. Furthermore, a similar abrupt decrease of the incidence rate of AGE was also observed at community level in the prospective cohort. Since these data are self-reported illness, they are not influenced by care seeking behavior, which strongly suggests a decrease in AGE incidence independent of healthcare seeking behavior. Similar sharp declines in AGE incidences in relation to COVID-19 barrier measures were also described in China^20^, Germany^21^, Great Britain^22^, the United States^23^ and Korea^24^.

It is biologically plausible that the barrier measures legislated by the government after the introduction of SARS-CoV-2 in France also had an impact on the transmission of the causative agents of AGE. Physical distancing and lockdowns have the potential to reduce direct transmission between persons, as well as transmission by contaminated surfaces^25^. Frequent handwashing, preferentially with soap and water, is one of the recommended barrier measures against AGE.

During the first lockdown, the combined effect of barrier measures along with closure of schools, bars and restaurants, and limitations of public and private social gatherings, would undoubtedly have limited the direct transmission of pathogens that cause AGE. Based on results from the CoviPrev surveys, it is not possible to assess individually the impact of each mitigation measure on AGE incidence as these measures were implemented simultaneously. However, the systematic adherence to handwashing, and no handshaking/kissing on the cheek/hugging shows relatively high frequencies during lockdown periods and beyond. While the frequencies of the systematic adherence to additional recommendations, including avoiding gatherings and staying at home as much as possible, were lower and varied through 2020, they still likely limited person-to-person transmission of gastrointestinal pathogens. For handwashing, the survey does not distinguish between handwashing using soap and water, or the use of alcohol based hand sanitizer. We must interpret the possible effect of this measure with caution. The use of hand sanitizers has been massively encouraged by health authorities worldwide to contain the SARS-CoV-2 spread but could have less impact on AGE than washing hands with water and soap^26,27^. Indeed, some alcohol based hand sanitizers might be ineffective against enveloped viruses such as norovirus, the main cause of AGE among humans, if the virucidal standards are not respected^28^.

The increase in AGE activity after the second national lockdown was more marked for children under 5 years of age compared to that observed for all age groups combined. During this second lockdown, child day-care facilities, kindergartens and schools remained open, but the barrier measures in preschool were less strict (no masks, no physical distance) than those in place in schools for children above 5 years of age. These differences could have favored viral transmission among children under 5 year. The third lockdown was slightly stricter than the second, and all child-daycare, preschool facilities and schools were closed. AGE activities, particularly in young children, remained at high levels after this 3rd lockdown and until July 2021, which coincides with a period of lower COVID-19 incidence, and a consequently overall decreased adherence to barrier measures.

This unusual high level in activities has already been described for other childhood diseases during the COVID-19 pandemic, particularly for bronchiolitis in France^17^ and Australia^29,30^. During the H1N1 pandemic in 2009, after a period of low AGE activity during the influenza pandemic peak, a resurgence in AGE incidence rate was observed, resulting in a winter peak of higher magnitude occurring 5 weeks later compared to the previous years^31^. As a result of COVID-19 barrier measures and lockdowns, two cohorts of children born in 2020 and 2021 were probably less exposed to rotaviruses and noroviruses, resulting in a lack of immune stimulation^32^ and therefore increasing the number of susceptible children. Consequently, there is a potential for an increase in AGE activity in young children after the lockdowns and following the expected decrease in adherence to barrier measures, once the pandemic is under control^33^. In addition, this resurgence may occur outside of the usual winter season.

Such a resurgence could have a profound impact on health services, in particular if high pediatric AGE caseloads coincide with increasing consultations for other pediatric infections such as bronchiolitis^17,29,30^. As the COVID-19 pandemic persists, and adherence to barrier measures will likely continue to vary depending on trends in COVID-19 incidence and government imposed measures, we may continue to observe unusual trends in AGE incidence. We highly recommend that the population remain vigilant in maintaining high adherence to preventive measures (particularly hand hygiene with soap and water, isolation at home when presenting AGE symptoms) in order to limit the transmission of AGE related pathogens.

## Data Availability

The data from the Sentinel Network, from SurSaUD, and CoviPrev are available as Open Data on the following websites:—Sentinel Network: https://www.sentiweb.fr/france/fr/?page=table.—SurSaUD (all ages): https://geodes.santepubliquefrance.fr/#view=map2&c=indicator.—CoviPrev: https://www.santepubliquefrance.fr/etudes-et-enquetes/coviprev-une-enquete-pour-suivre-l-evolution-des-comportements-et-de-la-sante-mentale-pendant-l-epidemie-de-covid-19. Data from SurSaUD for children under 5 years of age and GrippeNet.fr-COVIDnet.fr are available from the corresponding authors on request.
